# Integrated Geophysical and Hydro-chemical Investigations of Impact of the Ijemikin Waste Dump Site in Akure, Southwestern Nigeria, on Groundwater Quality

**DOI:** 10.5696/2156-9614-8.18.180604

**Published:** 2018-06-04

**Authors:** Oluwaseun E. Odipe, Rasheed A. Ogunleye, Musa Sulaiman, Suleiman S. Abubakar, Martins O. Olorunfemi

**Affiliations:** 1 Department of Geology, Obafemi Awolowo University, Ile-Ife, Nigeria; 2 Department of Environmental Health Sciences, School of Health, Allied and Environmental Science, College of Pure and Applied Sciences, Kwara State University, Malete, Kwara State; 3 Kano State Ministry of Water Resources and Rural Development, Kano State

**Keywords:** waste dump, leachate, geophysical investigation, hydro-chemical analysis, water quality

## Abstract

**Background.:**

Improper waste disposal can negatively impact the ecosystem and constitutes a major human health risk.

**Objectives.:**

The present study evaluated the environmental impact on groundwater quality of an open-air waste dump in Akure, southwestern Nigeria, using an integrated geophysical survey and hydro-chemical analysis of water samples.

**Methods.:**

The geophysical survey involved three dipole-dipole 2-D imaging profiles and seven vertical electrical soundings (VES) along three traverses. The dipole-dipole data were inverted using the Dipro for Windows software, while the VES data were quantitatively interpreted using partial curve matching and computer assisted 1-D forward modeling with the WINResist software. The VES interpretation results were used to generate geoelectric sections. For the hydro-chemical analysis, samples were taken from five hand-dug wells at various distances from the dumpsite. The samples were analyzed for temperature, pH, conductivity, total dissolved solids (TDS), and some major elements (calcium ion (Ca^2+^), magnesium ion (Mg^2+^), chloride ion (Cl^−^), nitrate (NO_3_^−^), sulfate (SO_4_^2−^)) whose concentration values were compared with World Health Organization (WHO) and Nigerian Industrial Standard (NIS) standards for assessment of groundwater quality.

**Results.:**

The VES curves revealed three distinct geoelectric/geologic layers with thicknesses and resistivities in the range of 0.7 - 2.0 m and 31 - 55 Ωm for topsoil, 6.2 - 14.6 m and 13 - 114 Ωm for the weathered layer, and a fresh basement with resistivity values ranging from 344 - ∞ Ωm.

In the hydro-chemical analysis, pH values ranged between 7.57 - 7.8, electrical conductivity ranged from 884 - 1510 μS/cm, and TDS ranged between 588 - 1008 mg/l. Concentration values of Ca^2+^ and Mg^2+^ ranged between 78 - 132 mg/l and 1.8 - 19 mg/l, respectively.

**Conclusions.:**

The results from the combined electrical resistivity methods showed relatively low resistivity values at the topsoil and weathered layers and the hydro-chemical assessment of water samples indicated that the topsoil and groundwater within the dumpsite may have been polluted by leachate.

**Competing Interests.:**

The authors declare no competing financial interests.

## Introduction

Improper waste disposal and management have led to the microbial and chemical contamination of the environment and water supply sources globally. There have been a number of successful investigations of the impact of leachate plume migration from waste dumps into the environment using various geophysical and hydro-chemical methods. 1, 2, 3, 4

The present study used integrated geophysical and hydro-chemical investigation methods to delineate the subsurface layers, thicknesses, and resistivity values, as well as the hydro-chemical characteristics of well water samples to assess the impact of leachate from the Ijemikin market waste dumpsite in Akure, southwestern Nigeria, on groundwater quality.

## Methods

The present study utilized integrated geophysical methods and a hydro-chemical analysis of water samples from wells around the Ijemikin waste dumpsite to investigate possible contamination of groundwater by leachate migration from the waste dump. The geophysical method employed in the present study was the electrical resistivity method involving the vertical electrical soundings (VES) technique with the Schlumberger array and 2-D imaging with the dipole-dipole array.

### Study area

**Figure 1:**
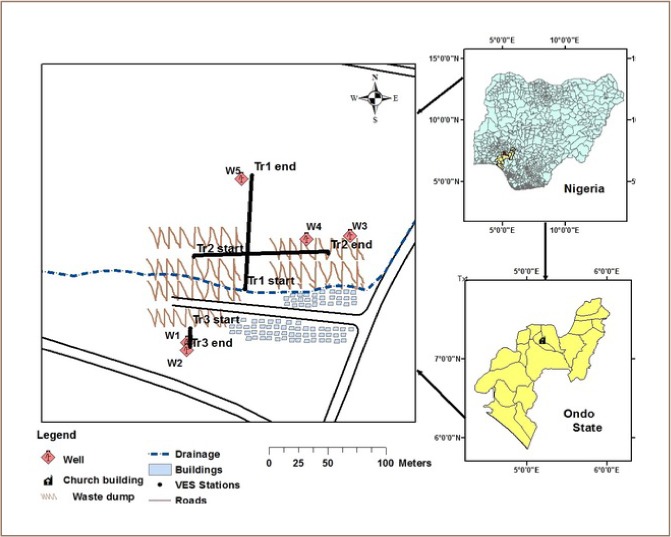
Map of the study area showing the traverses, VES stations and wells (Nigeria & Ondo State inset)

The study area, Ijemikin Market waste dumpsite, lies within latitude 7°14′48″ N to 7°14′58″ N and longitude 5°11′40″ E and 5°11′46″ E *(Figure 1)*. The area is underlain by the migmatite gneiss rock of the Precambrian basement complex rocks of southwestern Nigeria.^5^ The surface drainage shown in Figure 1 is a public concrete gutter system that runs through the community as a channel for storm water runoff.

Seven VES stations were investigated, while 2-D imaging was carried out along three traverses: two trending north-south and one trending east-west *(Figure 1)*. A dipole length of 5 m and expansion factor n varying from 1 to 5 was adopted.

### Geophysical methods

The VES data were processed by plotting the apparent resistivity values against electrode spacings (AB/2) on a bi-logarithmic paper as sounding curves. The curves were interpreted by partial curve matching and computer assisted 1-D forward modelling with WinRESIST version 1.0 software.^6^ The dipole-dipole data were inverted using the DIPRO software into the 2-D pseudosections and resistivity structures.

For the hydro-chemical test, five water samples collected from unlined hand-dug wells (without lining rings) within a radius of 60 m from the dumpsite were securely corked and stored in sample bottles which were thoroughly rinsed three times with the sample water on site before samples were taken and then transported to the laboratory for analysis within 24 hours. Physical parameters such as pH, temperature, and electrical conductivity were determined in situ using a portable digital conductivity and pH meter, while major cations (calcium ion (Ca^2+^), magnesium ion (Mg^2+^)), and anions (chloride ion (Cl^−^), nitrate (NO_3_^−^), sulfate (SO_4_^2−^)) were determined in the laboratory using standard methods. Calcium was determined using 0.05 M ethylenediaminetetraacetic acid (EDTA) titration and chloride by a standard solution of 0.005 M silver nitrate (AgNO_3_) argentometry titration using potassium chromate as an indicator. Bicarbonate and carbonates were determined by titration against a standard sulfuric acid (H_2_SO^4^) solution (0.0392 M). Magnesium was determined by atomic absorption spectroscopy (AAS) (Hanna, HI 98180). Sulfate was determined by direct reading using a spectrophotometer. Total hardness was computed using the formula equation: total hardness =2.5 Ca^2+^ + 4.1 Mg^2+^. The measured parameters were compared with the recommended water standards of the World Health Organization (WHO) and Nigerian Industrial Standard (NIS) to determine their qualities. 7, 8

**Abbreviations**
*NIS*: Nigerian Industrial Standard
*VES*: Vertical electrical soundings
*WHO*: World Health Organization

## Results

### Geophysical methods

**Figure 2:**
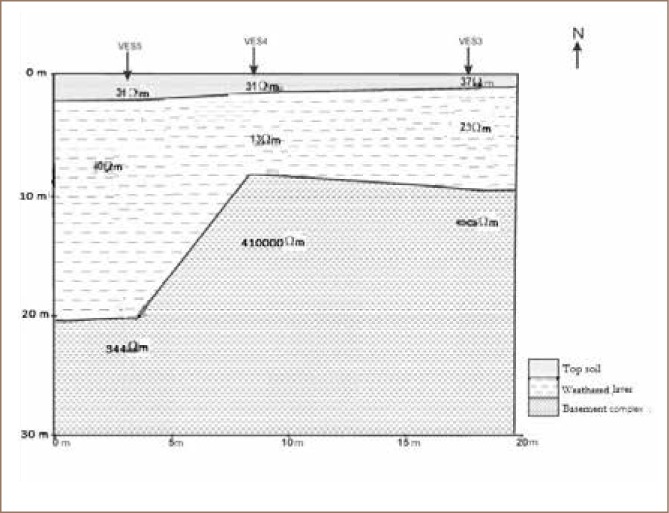
Geo-electric section showing VES stations 3, 4 and 5 along traverse 1

**Figure 3:**
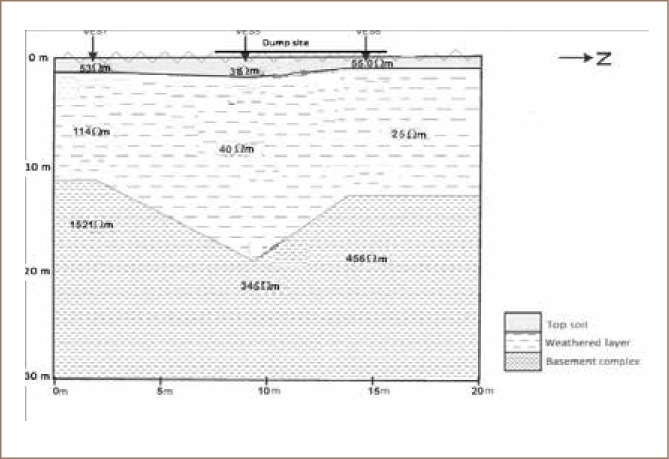
Geo-electric section showing VES stations 5, 6 and 7 along traverse 2

Geo-electric sections were developed by correlating the various VES points along the three traverses to reveal the subsurface layers, their thicknesses and resistivity values *(Figures 2 and 3)*.

#### Vertical electrical sounding

Three distinct geoelectric/geological layers (topsoil, weathered layer and fresh basement) were delineated.^9^

#### Dipole-dipole imaging

**Figure 4:**
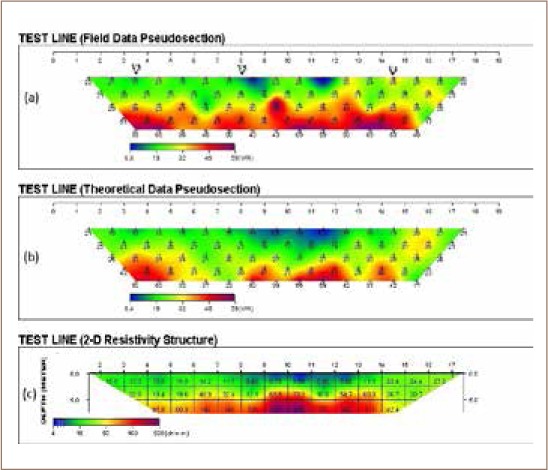
Dipole-dipole 2-D resistivity pseudosection along traverse 1

**Figure 5:**
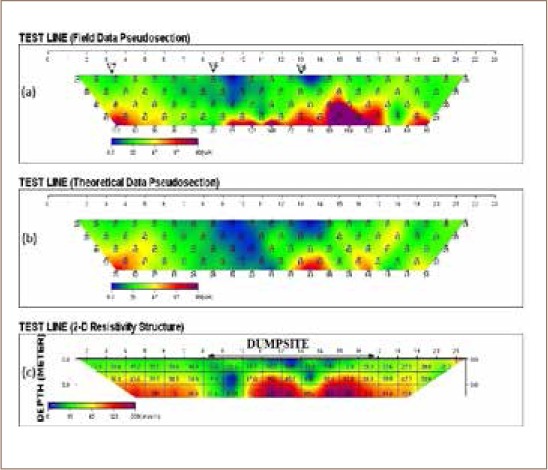
Dipole-dipole 2-D resistivity pseudosection along traverse 2

**Figure 6:**
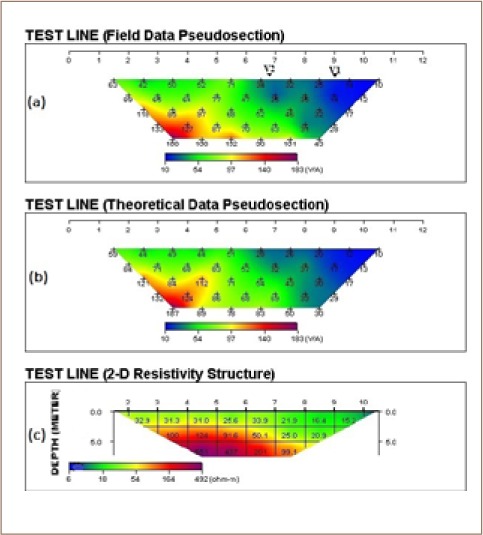
Dipole-dipole 2-D resistivity pseudosection along traverse 3

The 2-dimensional pseudosections and resistivity structures *(Figures 4, 5 and 6)* along the three traverses indicated showed variation in resistivity and thickness of the overburden above the presumably fresh basement.

#### Hydro-chemical results

A summary of the water quality results from the hydro-chemical analysis on the five groundwater samples taken from wells without lining rings within the study area are presented in Table 1.

**Table 1:** Hydro-chemical Parameters of Sampled Well Water Compared to WHO and NIS Recommended Standards

**Sample** **Description/Parameters**						**Water Quality Standards**	
	**Well 1**	**Well 2**	**Well 3**	**Well 4**	**Well 5**	**(WHO, 2004)^7^**	**(NIS, 2007)^8^**
***Total alkalinity***	200	220	280	170	190	200	ng
***Total acidity***	120	150	210	110	80	ng	ng
***Total hardness***	450	342	400	363	273	400	n/a
***Total dissolved solids (mg/l)***	828	711	978	588	1008	1000	500
***Conductivity (μS/cm)***	1236	1061	1470	884	1510	1000	1000
***pH***	7.78	7.57	7.86	7.80	7.78	6.50–8.50	6.50–8.50
***SO_4_^2−^ (mg/l)***	1.5	5.7	5.0	0.17	5.0	200	100
***NO^3−^ (mg/l)***	5.2	2.4	5.7	5.7	4.7	50	50
***HC0^3−^ (mg/l)***	240	264	336	204	228	ng	ng
***Cl^−^ (mg/l)***	122	83	146	79	69	250	250
***Mg^2+^ (mg/l)***	2	15	17	13	19	30	0.20
***Ca^2+^ (mg/l)***	177	112	132	124	78	75	ng

Abbreviations: ng, no guideline; n/a, not available.

## Discussion

### Geophysical methods

The combined geophysical methods revealed three distinctive geo-electric layers of topsoil, weathered layer and fresh basement, and identified low resistivity zones in the subsoil, which were indicative of leachate plume migration into the bedrock.^3^

#### Vertical electrical sounding

The geo-electric section along traverse 1 (*Figure 2*) showed evidence of low resistive topsoil with a resistivity value range of 31–37 Ωm and a weathered layer with resistivity values between 13 Ωm – 40 Ωm above a highly resistive basement complex with resistivity values ranging between 344–∞ Ωm. The geo-electric section along traverse 2 (*Figure 3*) revealed three geoelectric layers consisting of topsoil with low resistivity values ranging between 31–55 Ωm, and a weathered layer with resistivity values between 25–114 Ωm overlying a resistive basement complex with resistivity values ranging between 345–1521 Ωm. The relatively low resistivity values of less than 100 Ωm at the topsoil and weathered layer are indications of conductive materials which are suspected to be fluids and leachates from the waste dump in the subsoil.

#### Dipole-dipole imaging

Three resistivity structures along the three traverses shown in Figure 4c, 5c and 6c revealed 2-D subsurface imagery consisting of low resistive topsoil and weathered layers of resistivity values below 45 Ωm at a shallow depth of 5 m and a highly resistive underlying basement complex with a resistivity value above 123 Ωm. The very low resistivity areas around the dumpsite (*Figure 5c)* are possible indications of leachate or conductive fluids infiltrating into the subsurface from the overlying dumpsite.

### Hydro-chemical results

As shown in the hydro-chemical analysis results in Table 1, pH values ranged between 7.5 – 7.8 and were within the permissible range for safe water according to the WHO (2004)^7^ and NIS (2007)^8^ standards. The pH values can be classified as neutral to slightly alkaline. Electrical conductivity values ranged from 884–1510 μS/cm. All other well samples had conductivity values that were above the WHO and NIS threshold value of 1000 μS/cm as the maximum permissible safe level for potable water, except for Well 4. In addition, total dissolved solids (TDS) values ranged between 588 – 1008 mg/l which were all above the NIS threshold of 500 mg/l, but below the WHO threshold of 1000 mg/l, except for Well 5. The relatively high concentration values for electrical conductivity and TDS could be indicative of leachate pollution. The cation Ca^2+^ value for all wells ranged between 78 – 132 mg/l, which was higher than the WHO threshold of 75 mg/l.

## Conclusions

Hydro-chemical analysis of well water samples around the waste dump also examined environmental water quality and hydro-chemical parameter concentrations compared to established water quality standards.^7^, ^8^ Threshold values for drinking water quality revealed that the groundwater had been impacted by leachate from the dumpsite.^4^ The relatively low soil/subsoil resistivity values and concentrations of some hydro-chemical elements above permissible levels in the water samples indicate pollution of the soil/subsoil and groundwater by leachate from the investigated waste dump site.
